# Dr. Andrews

**Published:** 1861-03

**Authors:** 


DR. ANDREWS, Professor of Physiology in Queen's College, Birmingham, died of apoplexy, on the nineteenth of December.

